# 5.C. Skills building seminar: Learn how to contribute to raising awareness about public health in Europe with the EUPHW

**DOI:** 10.1093/eurpub/ckac129.285

**Published:** 2022-10-25

**Authors:** Organised by: EUPHA, Chair persons: Annemieke Schuffelen (EUPHA), Dineke Zeegers Paget (EUPHA)

## Abstract

The European Public Health Association (EUPHA) aims to bring together the public health workforce for professional exchange and collaboration through Europe. EUPHA is always looking for new tools and that is how the European Public Health Week (EUPHW) started: it promotes collaboration among the public health community in Europe and raises awareness about public health. The EUPHW has been an annual initiative since 2019, inspired by similar initiatives in Austria and the USA. The EUPW cannot be a success without the public health professionals who organise their own public health event. EUPHA needs those enthusiasts! And therefore, this workshop aims to encourage individuals to organise an event during the next European Public Health Week. For event hosts, the EUPHW is the perfect opportunity to share your expertise and contribute locally, regionally and nationally to topics of great relevance all across Europe. We are very well aware that organising an event may be daunting which is why EUPHA is organising this workshop. Over the years we built a network and created materials for promotion, which will make it easier to organise an event. In this workshop, we would like to give you the tools for organising an event during the EUPHW. The EUPHW has a rich portfolio: the past four editions of the EUPHW recorded approximately 550 online and in-person activities, hosted by more than 40 different countries in multiple languages. In this workshop, we will let members of EUPHW's steering committee share their favourite activities from the past few EUPHW editions. What made those events successful? We will talk about engagement with the future generation of public health professionals (EUPHAnxt) and building continuity and sustainable partnerships. Hosts will share their experiences of the EUPHW. What is their perspective on organising a public health week event at both national and European levels? How does the day-to-day coordination work? This sharing of knowledge will be followed by interaction. Together, we will imagine the European Public Health Week of 2023: what topics are important for public health professionals? On what subject do you want to make the case and amplify existing messages? What collaborations do you see? What kind of event would you like to organise? The topics that will be covered include choice of themes and key messages, logistics, target audiences, governance, communication, partnerships, stakeholder engagement, as well as involvement types and evaluation.

The EUPHW is supported by the WHO Regional Office for Europe.

**Key messages:**

• The European Public Health Week offers a unique opportunity to showcase public health, raise awareness about important health themes and promote collaboration among the public health community.

• This workshop aims to inspire individuals to get involved in the European Public Health Week, and contribute locally to topics of great relevance across the European Region.

